# A special issue on calcium dynamics of the heart: remodeling of ion channels and regulatory pathways

**DOI:** 10.1007/s00424-021-02532-3

**Published:** 2021-03-05

**Authors:** Dan J. Bare, Lixia Yue, Xun Ai

**Affiliations:** 1 Department of Physiology and Biophysics, Rush University Medical Center, Chicago, IL 60612 USA; 2 Department of Cell Biology, Calhoun Cardiology Center, University of Connecticut Health Center, Farmington, CT 06030 USA

**Fig. 1:**
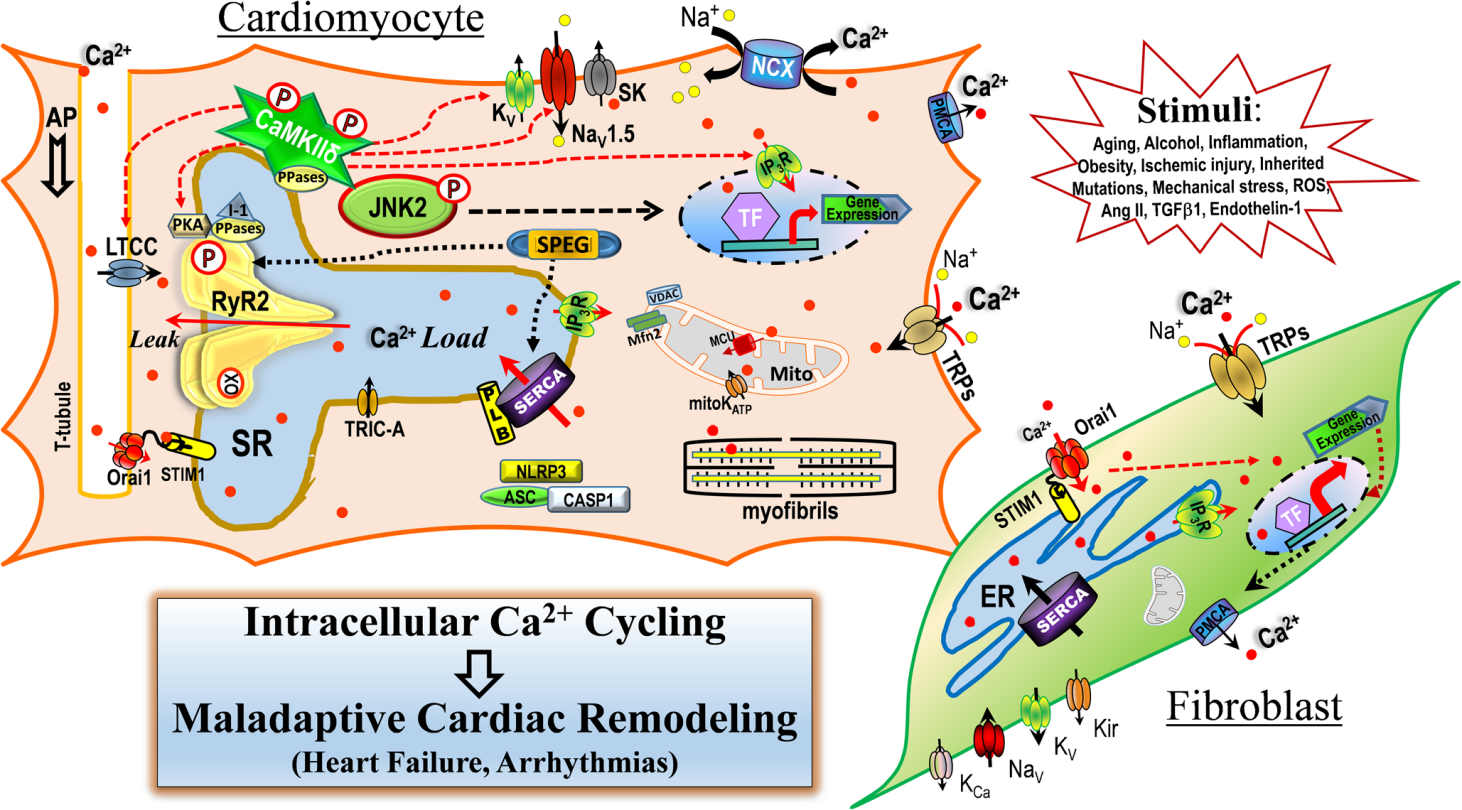
Schematic diagram of demonstrated mechanisms of intracellular Ca^2+^ cycling-mediated cardiac maladaptive remodeling which could lead to various types of cardiac functional abnormalities such as heart failure and arrhythmias

Calcium (Ca^2+^) has been identified as an essential regulator of cardiac function since 1883 [16]. To meet the ever-changing metabolic and energy demands placed on the heart by the body, there is a strictly synchronized control of coupling between excitation and contraction, which is defined by sequential dynamics of Ca^2+^ signaling that links the cell membrane depolarization, ion channel activation, and the events of Ca^2+^-induced-Ca^2+^-release from the sarcoplasmic reticulum Ca^2+^ store to the activation of the myofibrillar contractile machinery during each heartbeat. A balanced intracellular Ca^2+^ homeostasis is maintained by highly coordinated actions of the intracellular Ca^2+^ cycling machinery including Ca^2+^ channels, Ca^2+^ pumps/transporters located on the plasma membrane, endo/sarcoplasmic reticulum (ER/SR) and mitochondrion in myocytes and fibroblasts [1, 4, 9]. In this *Special Issue*, we compiled a coherent set of review and original research articles to introduce the major advances regarding our current understanding of the cardiac Ca^2+^ signaling via ion channels including voltage-gated Ca^2+^ channels, ligand-gated Ca^2+^ channels, ryanodine receptors (RyR2s), inositol 1,4,5-trisphosphate receptors (IP_3_Rs), Ca^2+^ pumps/transporters, and transient receptor potential (TRP) channels (Fig. 1) under both physiological and pathological conditions. Moreover, translational studies aimed at developing novel therapeutic strategies to prevent and treat patients with heart failure and cardiac arrhythmias were also discussed.

In this *Special Issue*, several articles (Blatter et al., Lahiri et al., McKee et al., Hamilton et al., Wang et al., Xie et al., Rosenberg et al., Fill et al.) [2, 7, 8, 12, 13, 17–19] have thoroughly reviewed the physiological role of the excitation-contraction coupling (ECC) in the cardiomyocyte and a well-established concept that defective intracellular Ca^2+^ cycling acts as a key contributing factor to an elevated predisposition for arrhythmias and/or impaired cardiac contractility in pathologically altered hearts. Under certain conditions such as exercise, aging, excessive alcohol exposure, obesity, diabetes, inherited mutational burdens, ischemia, systemic inflammation, these extrinsic/intrinsic stress stimuli trigger adaptive remodeling of Ca^2+^ handling machinery and regulatory signaling pathways in cardiac cells that are necessary to deliver an adequate acute response but also ensures long-term survival. Unfortunately, sustained stimulation of these stress stimuli ultimately disrupts the normal dynamics of cardiac Ca^2+^ handling, which results in myocardial maladaptive remodeling toward cardiac dysfunction. In addition, disrupted intracellular Ca^2+^ cycling and SR Ca^2+^ mishandling critically contribute to the development of fatal ventricular fibrillation (a major cause of sudden cardiac death) and atrial fibrillation (the most common arrhythmia, with a higher mortality due to significantly increased risk for stroke and heart failure). Moreover, detailed discussions by Lahiri et al., McKee et al., Hamilton et al., Wang et al., Rosenberg et al., [8, 12, 13, 17, 18] were also included regarding the new findings of regulatory mechanisms of stress stimuli (i.e., aging, excessive alcohol abuse, inflammation, ischemic-reperfusion) or inherited gene mutations (i.e., Catecholaminergic Polymorphic Ventricular Tachycardia (CPVT) and Long QT Syndrome (LQTS)) in disrupted SR Ca^2+^ dynamics via altered channel function of RyR2, IP_3_R2, and STIM1-Orai1, which lead to changed SR Ca^2+^ load and significantly increased diastolic SR Ca^2+^ leak promoting the initiation or progression of arrhythmias and heart failure. Altered enzyme activity and/or changed interactions between kinases/protein phosphatases and ion channels critically contribute to these stress-evoked changes in the Ca^2+^ homeostasis and cardiac electrical and mechanical dysfunction. While hyperactivation of CaMKIIδ is well-known to promote maladaptive cardiac remodeling during disease onset and progression, recent studies have uncovered crucial roles of several important kinases and protein phosphatases including stress-response kinase JNK2, striated muscle preferentially expressed protein kinase (SPEG), protein phosphatases PP1, PP2A, and endogenous PP1 inhibitor protein (I-1) in intracellular Ca^2+^ cycling and SR Ca^2+^ dynamics as well as enhanced arrhythmogenicity in the stressed heart. These newly identified channel modifiers within the macro-molecular complex of the Ca^2+^ handling machinery suggest the complexity of the regulation of the ion channel activities and intercellular Ca^2+^ homeostasis in cardiac myocytes.

The beat-to-beat Ca^2+^ signaling event occurs in both atrial and ventricular cells but with some notable functionally relevant and structurally based differences. Blatter et al. [2] provide a timely review regarding the distinctive mechanisms of ECC in the atria and ventricles, while the functional role of an “auxiliary” pathway of Ca^2+^ release involving the less studied cardiac IP_3_R type 2 (InsP_3_R-2) Ca^2+^ release channel for atrial ECC and atrial alternans in arrhythmogenesis were also presented. Moreover, accumulating studies suggest that mitochondrial Ca^2+^ and the mitochondrial Ca^2+^ handling units such as mitochondrial Ca^2+^ uniporters (MCU) are also involved in physiological Ca^2+^ homeostasis as well as pathological intracellular Ca^2+^ mishandling in diseased hearts. Ernst et al. [5] reported a new approach to assess the coupling between mitochondrial Ca^2+^ and cytosolic Ca^2+^ dynamics using genetically-encoded Ca^2+^ indicators (GECIs) in human-induced pluripotent stem cell-derived cardiomyocytes (hiPSC-CMs) and adult cardiomyocytes (ACMs). It should be noted that cardiac maladaptive function during the process of pathological myocardial remodeling (i.e., HF, AF) is well known to result in structural and biochemical changes related to the complex alterations of ion channel function, protein abundance, and protein post-translational modifications. Biochemical assays and proteomic analyses have thus been commonly employed. Rennison et al. [15] reported studies using a proteomic approach on the left atrial appendage from AF patients undergoing mitral valve repair and Maze surgery, showing significant expression changes in proteins related to mitochondrial energy production, which could contribute to cardiac contraction and electrophysiological remodeling in promoting AF pathogenesis. In addition, Yang et al. [20] also reported alterations of several housekeeping proteins in human heart tissue that were heart chamber-specific and highly disease context dependent in their expression levels. Past genetic studies have suggested that a strong heritable component is associated with the risk of AF and is inclusive of genes related to transcription factors. Due to the complexity of the etiology and progression of cardiac diseases, contributions of expression of genes and proteins in pathological Ca^2+^ mishandling and cardiac dysfunction are clearly worthy of future investigation. A much better understanding of the complex genetic regulatory networks involved in the cardiac pathogenesis will be critical for developing novel personalized therapies for patients.

In addition to the critical regulation of ECC, excitation-transcriptional coupling, and various other intracellular signaling cascades in cardiac contractile dysfunction and cardiac arrhythmia development, other ion channels including Ca^2+^-activated K^+^ channels and TRP channels also contribute to the intracellular Ca^2+^ homeostasis. Ca^2+^-activated small conductance K^+^ channels, SK, or apamin-sensitive SK (I_KAS_) are differentially expressed in the heart. Zhang et al. [21] presented an excellent review covering the discovery of SK channels in the heart to the most recent update in the understanding of the roles of SK channels in arrhythmias and HF. Chen et al. [3] concisely summarized how I_KAS_ channels are regulated and are involved in arrhythmias and proposed that sexual dimorphism in autonomic control of I_KAS_ plays a role in *J* wave syndrome. In contrast to the SK channel, the Ca^2+^-activated TRPM isoform 4 (TRPM4) is a non-selective monovalent cation channel impermeable to Ca^2+^ ions. Dysfunction of TRPM4 contributions to electrophysiological abnormalities under a variety of inherited cardiac conduction block conditions and Brugada syndrome and is also associated with various forms of acquired heart defects such as arrhythmias and heart failure. Hu et al. [10] elegantly demonstrated how TRPM4 contributes to early afterdepolarization (EAD) and promotes arrhythmogenicity via CaMKII-dependent abnormal Ca^2+^ homeostasis. Feng et al. [6] provided new evidence suggesting that TRPM4 significantly upregulated in cardiac fibroblasts in heart failure patients; however its contribution in enhanced arrhythmogenicity in heart failure requires further investigation. Medert et al. [14] knocked down TRPM4 in adult hearts by using a myocyte-targeted AAV9-RNAi approach, providing a nice tool for future investigation regarding the functional role of TRPM4 in the heart. Whereas the ionic homeostasis of cardiac cells is finely tuned by multiple ion channels, transporters, and ionic exchangers in the plasma membrane, the ionic homeostasis of intracellular organelles is also delicately maintained by influx and efflux of different ions. Although it remains debatable in this research field, the potential contribution of the TRIC-A channel in mediating K^+^ influx to ER/SR to counter-balance Ca^2+^ release was thoroughly reviewed by Zhou et al. [22]. Not only is the ionic homeostasis in cardiac cells essential for normal heart function, but abnormal extracellular ionic concentrations resulting from pathological conditions such as ischemia and renal failure could also promote arrhythmias, which indicates the need for further investigation. In addition, King et al. [11] nicely demonstrated how the combined effects of Na^+^ and Ca^2+^ can differentially modulate conduction during hyperkalemia and how enhancing determinants of ephaptic coupling may attenuate conduction changes in various physiologic conditions.

Over the past few decades, significant progress has been made in understanding the underlying mechanisms by which intracellular Ca^2+^ cycling mediates various types of cardiac functional abnormalities such has heart failure and arrhythmias. This *Special Issue* highlights some of the major progresses that have been made regarding changes of intracellular Ca^2+^ homeostasis via ion channels regulated by either Ca^2+^-induced SR Ca^2+^ release or a number of ion channel modifiers including kinases and protein phosphatases in response to various intrinsic or extrinsic stimuli. All of these exciting new perspectives lay the groundwork for future investigation such as the inter-relationship between kinases and protein phosphatases as well as between organelles in calcium handling and homeostasis in both normal function and in the pathological state. The complexity of the genetic regulatory networks in the dysfunction of intracellular cycling and maladaptive cardiac remodeling and disease onset and progression are also worthy of further investigation. Moreover, the interactions between SR, mitochondrion, and nucleus in orchestrating normal intracellular Ca^2+^ homeostasis as well as aberrant Ca^2+^ handling under pathological conditions are an interesting research direction deserving more attention. While the functional role of fibroblasts in cardiac electrophysiology and structural remodeling has recently emerged as a research point of interest, the interaction between myocytes and fibroblasts is clearly another new research direction in need of attention. These novel discoveries and new directions in future research will hopefully lead to targeted and effective therapies to prevent the maladaptive remodeling and disease progression in the heart.
